# Affective priming in major depressive disorder

**DOI:** 10.3389/fnint.2012.00076

**Published:** 2012-10-05

**Authors:** Joelle LeMoult, K. Lira Yoon, Jutta Joormann

**Affiliations:** ^1^Department of Psychology, University of MiamiCoral Gables, FL, USA; ^2^Anxiety Treatment and Research Centre, St. Joseph's HealthcareHamilton, ON, Canada; ^3^Department of Psychology, University of MaineOrono, ME, USA

**Keywords:** affective priming, cognitive biases, depression

## Abstract

Research on cognitive biases in depression has provided considerable evidence for the impact of emotion on cognition. Individuals with depression tend to preferentially process mood-congruent material and to show deficits in the processing of positive material leading to biases in attention, memory, and judgments. More research is needed, however, to fully understand which cognitive processes are affected. The current study further examines the impact of emotion on cognition using a priming design with facial expressions of emotion. Specifically, this study tested whether the presentation of facial expressions of emotion affects subsequent processing of affective material in participants with major depressive disorder (MDD) and healthy controls (CTL). Facial expressions displaying happy, sad, angry, disgusted, or neutral expressions were presented as primes for 500 ms, and participants' speed to identify a subsequent target's emotional expression was assessed. All participants displayed greater interference from emotional vs. neutral primes, marked by slower response times to judge the emotion of the target face when it was preceded by an emotional prime. Importantly, the CTL group showed the strongest interference when happy emotional expressions served as primes whereas the MDD group failed to show this bias. These results add to a growing literature that shows that depression is associated with difficulties in the processing of positive material.

Cognitive theories of depression propose that individuals with Major Depressive Disorder (MDD) show mood-congruent cognitive biases, typically giving preference to the processing of negative vs. positive material. According to Beck (1967), negative biases in MDD stem from stable cognitive patterns, termed schemata, which serve as negative filters through which individuals view themselves, the world, and others. Cognitive theories further highlight the interconnectedness of negative schemata in depression, which leads to automatic and efficient processing of mood-congruent information (Ingram, 1984). Thus, the presence of a negative stimulus will easily activate other negative thoughts and memories in depression. It is this high degree of interconnectedness of negative vs. positive information that is believed to heighten risk for the onset and maintenance of depressive episodes (e.g., Ingram, 1984; Taylor and Ingram, 1999), highlighting the importance of examining the effect of emotion on cognition.

The interconnection of mood-congruent material has been tested using affective priming designs (e.g., Fazio et al., 1986; Murphy and Zajonc, 1993). In a typical affective priming experiment, two words (referred to as *prime* and *target*) are presented sequentially, and participants are asked to indicate the valence of the target as quickly as possible. The speed with which people respond to the target is believed to be dependent on the prime. People typically demonstrate a priming effect: they process the target faster when the prime is valence-congruent (e.g., both prime and target are positive) compared to when the prime valence-incongruent (e.g., prime is neutral but target is positive). Priming occurs because the prime activates other information of the same valence (e.g., Fazio et al., 1986). The more closely interconnected valence-congruent information, the faster people will respond to the target. Previous studies have shown that the presentation of a prime can affect feelings, thoughts, and action tendencies (e.g., Neely, 1991; Bargh et al., 1996).

Given that negative information is more closely interconnected in depression, cognitive theories propose that depressed compared to control participants will show stronger priming for negative stimuli; however, priming studies in depression have not yielded consistent results. Bradley et al. (1995), for example, used a lexical decision task with subliminal primes, and found greater priming for negative words in depressed vs. control participants. In contrast, Matthews and Southall (1991) did not find priming from negative words in depression despite using a similar design. Null findings have also been reported in several other studies using subliminal primes (Koschack et al., 2003; Dannlowski et al., 2006a). Interestingly, Dannlowski et al. (2006b) found evidence of reverse priming, or interference. The authors examined the impact of prime words on how quickly participants judged the valence (positive or negative) of the targets. Rather than responding faster on congruent prime-target trials, depressed participants were slower to judge the valence of negative target words following negative primes. Taken together, studies do not provide consistent evidence of enhanced priming of negative concepts in depression, which stands contrary to predictions made by cognitive theories of depression.

Difficulties obtaining priming effects could be related to the length of time the prime is presented. Priming studies in depression traditionally have examined the automatic activation of schemata by presenting primes subliminally. Many studies on cognitive processing in depression, however, have reported that biases are more consistently found at later stages of processing (e.g., see Mathews and MacLeod, 2005, for a review). With this in mind, to better understand cognitive biases in MDD, stimuli may have to be presented for longer durations to allow more elaborative processing of the material. The current study, therefore, examined how emotional information presented for a prolonged period of time affected the processing of subsequent emotional targets.

In addition, the choice of stimuli may affect priming effects in depression. Thus far, the majority of studies have utilized word stimuli (e.g., Bradley et al., 1995; Dannlowski et al., 2006b). Given the importance of deficits in social functioning in the maintenance and recurrence of depression (Blair, 2003), facial expressions may provide stronger and more relevant stimuli than words. The ability to quickly and accurately identify facial expression of emotions is critical for successful interpersonal functioning (e.g., Hess et al., 1988). Increasing evidence suggests important differences in the way depressed and control participants process facial expressions. In fact, results from both behavioral and neuroimaging studies highlight that depressed individuals differ from healthy controls (CTL) in the processing of facial expressions. Some studies have reported that depressed participants exhibit global deficits when processing facial expressions of emotion (e.g., Feinberg et al., 1986), whereas others indicate that MDD is associated with difficulty identifying specific emotions (e.g., happy but not sad; Suslow et al., 2001). Moreover, results from neuroimaging studies indicate that, compared to healthy controls, depression is associated with a different pattern of neural responses to happy vs. sad expressions (e.g., Surguladze et al., 2005) as well as differential neural responses to facial expression of disgust (e.g., Surguladze et al., 2010). Given the substantial evidence suggesting the importance of facial processing in the severity, persistence, and relapse of depressive episodes (e.g., Hale, 1998; Bouhuys et al., 1999a,b), it might be particularly relevant to examine priming effects using facial expressions of emotion.

The current study used a priming design to test the impact of emotion on participants' speed to cognitively process subsequent information. Emotional primes were presented for a prolonged period of time to allow thorough processing, and we tested the impact of these primes on participants' speed to identify a subsequent congruent or incongruent target. We predicted that all individuals would show priming effects, evidenced by faster processing of targets when the prime is of congruent valence compared to a neutral prime. However, we expected depressed participants, compared to healthy controls, to show especially strong priming effects for negative vs. positive material, thereby highlighting the enhancing effect of negative emotions on processing speed for MDDs but not CTLs.

## Methods

### Participants

Adults between the ages of 18 and 60 were recruited via newspaper advertisements and Internet postings. Potential participants were screened over the phone for initial inclusion and exclusion criteria. Two groups of individuals were included in the study: participants who met DSM-IV criteria for MDD and participants who did not meet criteria for a current or past Axis I disorder (CTL). Individuals were excluded if they had severe head trauma, a learning disability, or met DSM-IV criteria for bipolar disorder, alcohol abuse, or substance abuse with the past 6 months. After participants provided informed consent, diagnoses, and exclusion criteria were confirmed in the laboratory by trained and experienced interviewers using the Structured Clinical Interview for DSM-IV (SCID; First et al., 1996). Based on the SCID, 19 MDD and 30 CTL participants were deemed eligible and included in the study.

### Affective priming task

#### Stimuli

Pictures were selected from the Karolinska Directed Emotional Faces (Lundqvist et al., 1998), which consisted of black-and-white photographs of Caucasian individuals portraying a variety of facial expressions. For the current study, we selected 29 male and 28 female faces depicting neutral, happy, sad, angry, and disgusted expressions. Pictures of 11 additional models were used during the practice trials. All pictures were cropped just below the chin, above the hairline, and at the start of each ear. Pictures were approximately 8 cm × 10 cm in size and each pair was presented approximately 14 cm apart (measured from their centers).

#### Design

In the priming task (adapted from Yoon and Zinbarg, 2007), photograph pairs were presented sequentially on the computer screen using eprime software. Each trial began with “Ready” displayed for 500 ms in white text in the center of a black computer screen. Next, the first photograph (*the prime*) was presented in the center of the screen for 500 ms. Participants were asked to attend to the prime photograph, but they did not need to take any action. Immediately following the offset of the prime, a second photograph (*the target*) of a different actor was displayed in the center of the screen. The target photograph remained on the screen until participants judged the valence (positive or negative) of the facial expression using the 1 and 2 keys of a standard keyboard's number pad. Participants' reaction time to judge the valence of the target face was used to calculate the priming score (see below). The key 1 was partially covered with a sticker labeled N for negative valence, and the key 2 was partially covered with a sticker labeled P for positive valence. Participants were asked to use these keys to make their valence ratings as quickly and as accurately as possible. To test our hypothesis, it was critical to ensure that participants were consciously processing the prime and target facial expressions. For that reason, after both photographs went off the screen, participants saw a screen displaying the question, “Which picture was friendlier?” The accuracy of participants' response provided a check of their attention to both photographs. Participants then judged whether the first or second face was friendlier. Participants used the 1 and 2 keys of the number pad to make their friendly judgment: 1 indicated the first face was friendlier, and 2 indicated the second face was friendlier. Participants completed 10 practice trials before going on to complete 240 task trials.

The target faces depicted happy, sad, angry, or disgusted emotional expressions. For each emotional category, 20 trials were emotion-emotion (the prime and target displayed the same emotional expression), 20 were neutral-emotion (the prime depicted a neutral expression but the target depicted an emotional expression), and 20 were fillers (the prime and target depicted two different emotional expressions). Filler trials were included in the design so that emotional primes would not lead to participants anticipating a target of congruent valence. Given that filler trials consisted of a different number of each prime-target pair type (e.g., angry-sad vs. angry-disgust, vs. angry-happy) for each participant and that we did not expect participants to respond to negative-negative and negative-positive pair types the same, we did not include filler trials in the analyses.

### Questionnaires

#### BDI

To measure depression severity at the time when the priming task was administered, participants completed the Beck Depression-Inventory-II (BDI-II, Beck et al., 1996). This 21-item self-report measure of the severity of depressive symptoms has shown excellent reliability and validity (α = 0.92; Beck et al., 1996).

### Procedure

Participants first came into the laboratory to partake in the SCID, which took approximately 2 h. Eligible participants were scheduled for their second session, which took place typically within 2 weeks of the SCID. During the second session they completed the priming task and the BDI. The current study was approved by the University of Miami Internal Review Board (IRB).

## Results

### Participant characteristics

Clinical and demographic characteristics of the participant groups were examined (see Table 1). First, BDI scores were analyzed to confirm that MDD participants continued to exhibit high levels of depression at the time the priming task was completed. Given that two participants in the MDD group had BDI scores less than 10, these participants were excluded from further analysis. We analyzed data on the remaining 30 CTL and 17 MDD participants. Percent female did not differ between the CTL and MDD groups, χ^2^(1, N = 47) = 0.64. Race/ethnicity also did not differ across the two groups, χ^2^(4, N = 45) = 4.85. Ethnicity data are missing from two participants who elected not to provide this information. In addition, age did not differ in the CTL and MDD groups, *t*_(45)_ = 0.86, all *p* > 0.05. There was, however, the anticipated significant difference in participants' BDI scores, *t*_(45)_ = 11.11, *p* < 0.001, with CTL participants obtaining significantly lower BDI scores than MDD participants. Based on the SCID, 10 of the MDD participants also met criteria for one or more DSM-IV disorder, including panic disorder with or without agoraphobia, agoraphobia without panic disorder, social phobia, specific phobia, obsessive-compulsive disorder, posttaumatic stress disorder, and generalized anxiety disorder.

**Table 1:** **Participant characteristics**.

**Variable**	**Group**	
	**CTL**	**MDD**
Percentage of women	46.67	58.82
Percentage of race/ethnicity	−	−
American Indian or Alaska native	3.45	0.00
Black or African American	31.03	6.25
White—Non Hispanic or Latino	17.24	31.25
White—Hispanic or Latino	41.38	50.00
Other	6.90	12.50
Age (SD)	37.17 (12.71)	40.59 (13.66)
BDI (SD)	3.13 (4.57)^a^	29.29 (11.46)^a^
**# WITH COMORBID AXIS-I DISORDERS**		
Panic disorder with or without agoraphobia	−	5
Agoraphobia without panic disorder	−	1
Social phobia	−	3
Specific phobia	−	6
Obsessive-compulsive disorder	−	1
Posttraumatic stress disorder	−	2
Generalized anxiety disorder	−	4

Note: CTL, control; MDD, major depressive disorder; BDI, Beck Depression Inventory-II; participants may have more than one comorbid diagnosis.

a p < 0.001.

### Attention check

In order to adequately test our hypotheses, it was critical to ensure participants were consciously processing the prime and target facial expressions. To check for this, the accuracy with which participants judged the relative friendliness of facial expressions was examined on trials in which neutral facial expressions served as primes. The frequency that participants judged the emotional facial expression as more friendly was compared across happy, sad, angry, and disgusted trials. Friendly ratings are missing for five participants for whom there was a computer error. A two-way group (MDD, CTL) by emotion (happy, sad, angry, and disgusted) repeated-measures analysis of variance (ANOVA) was conducted on number of times the emotional face was judged friendlier than the neutral face. The main effect of group was not significant, *F*_(1, 40)_ = 0.80, *p* > 0.05, η^2^ = 0.02. However, there was a significant main effect of emotion, *F*_(3, 120)_ = 325.23, *p* < 0.001, η^2^ = 0.89, see Table 2. As expected, happy faces were judged friendlier more often than sad faces, *t*_(41)_ = 23.64, angry faces, *t*_(41)_ = 24.81, and disgusted faces, *t*_(41)_ = 21.00, all *p* < 0.001. In addition, sad faces were judged friendlier more often than angry faces, *t*_(41)_ = 3.61, and disgusted faces, *t*_(41)_ = 4.84, both *p* < 0.01. In contrast, there was no difference in the frequency with which participants judged angry vs. disgusted faces friendlier, *t*_(41)_ = 1.90, *p* > 0.05. The group by emotion interaction also did not reach significance, *F*_(3, 120)_ = 0.37, *p* > 0.05, η^2^ = 0.01. Thus, the accuracy with which participants attended to both facial expressions did not differ by group.

**Table 2:** **Percent trials emotional face judged friendlier than neutral face as a function of emotion and group**.

**Emotion (%)**	**Group**	
	**CTL**	**MDD**
Happy^a^	92.15 (12.90)	92.50 (14.55)
Sad^a^^,^^b^	18.35 (14.90)	22.50 (14.20)
Angry^a^^,^^b^	12.85 (10.80)	12.50 (17.90)
Disgusted^a^^,^^b^	8.00 (8.25)	12.90 (18.05)

a Happy faces were judged friendlier more often than sad faces, angry faces, and disgusted faces, p_s_ < 0.01.

b Sad faces were judged friendlier more often than angry faces and disgusted faces, p_s_ < 0.01.

### Priming

To assess priming, reaction times to indicate the target face valence were examined depending on whether the prime displayed a congruent or neutral facial expression. A priming score was calculated for each emotional expression using the following equation (see Table 3 for means by trial type):

Priming Score =RT(neutral-emotion)−RT(emotion-emotion)

Neutral-emotion indicates the prime displayed a neutral expression; emotion-emotion indicates the prime displayed the same emotional expression as the target. Therefore, RT (neutral-emotion) indicates the mean reaction time to indicate the valence of the target face when the prime is neutral. According to this formula, larger priming scores indicate people were faster when prime-target pairs had congruent facial expressions. Response times from only correct responses were examined. Error rates were low (less than 10% across all participants). To eliminate outliers, reaction times exceeding plus or minus one standard deviation from the mean were eliminated. Percent outlying trials did not differ in the CTL (10.82%) and MDD groups (8.87%), *t*_(45)_ < 1, *p* > 0.05.

**Table 3:** **Priming data by emotion and trial type**.

**Emotion**	**Reaction time (in ms)**	
	**Emotion–emotion**	**Neutral–emotion**
**HAPPY**		
CTL	1,559.91 (468.26)	1,276.69 (308.09)
MDD	1,385.42 (415.96)	1,176.28 (381.25)
**SAD**		
CTL	1,657.47 (492.67)	1,628.20 (411.99)
MDD	1,466.51 (570.49)	1,434.44 (376.02)
**ANGRY**		
CTL	1,701.37 (469.24)	1,588.27 (402.61)
MDD	1,446.50 (426.11)	1,346.19 (398.14)
**DISGUSTED**		
CTL	1,618.84 (467.57)	1,567.35 (364.22)
MDD	1,492.42 (496.87)	1,282.10 (339.46)

Note: Standard deviations in parentheses. CTL, control participants; MDD, participants diagnosed with Major Depressive Disorder.

**Figure 1:**
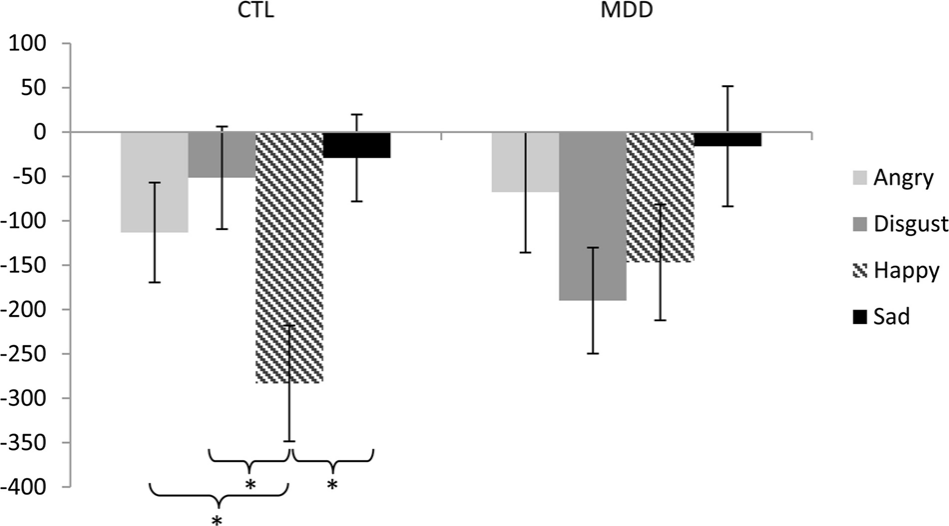
**Priming scores for each emotion (angry, disgust, happy, and sad) for both control (CTL) and depressed (MDD) participants**. Negative priming scores reflect reverse priming or difficulty disengaging from the emotional compared to neutral primes. Error bars indicate ±1 SE. ^*^ p < 0.02.

To test our main hypothesis regarding depression and priming from emotional facial expressions, a two-way group (MDD, CTL) by emotion (happy, sad, angry, and disgusted) repeated-measures ANOVA was conducted on priming scores. The ANOVA yielded a significant main effect of emotion, *F*_(3, 135)_ = 5.62, *p* < 0.001, η^2^ = 0.11, and a significant group by emotion interaction, *F*_(3, 135)_ = 2.88, *p* < 0.04, η^2^ = 0.06; see Figure 1. The main effect of group was not significant, *F*_(1, 45)_ = 0.04, *p* > 0.05, η^2^ = 0.00. Overall, participants displayed a *negative* priming score that suggests reverse priming. Reverse priming scores significantly differed from zero for happy, *t*_(46)_ = 4.83, angry, *t*_(46)_ = 2.24, and disgust faces, *t*_(46)_ = 2.34 *p* < 0.05; however, reverse priming scores did not significantly differ from zero for sad faces, *t*_(46)_ < 1, *p* > 0.05. Between-group follow-up analyses revealed no significant difference between the CTL and MDD groups' scores for happy, sad, angry, or disgusted faces [*t*_(45)_ = 0.50, 1.55, 1.37, and 0.16 respectively, all *p*_*s*_ > 0.05]. Within-group follow-up analyses revealed a significant main effect of emotion for the CTL group, *F*_(3, 87)_ = 7.59, *p* < 0.001, η^2^ = 0.21. Specifically, CTLs demonstrated greater reverse priming for happy faces than angry *t*_(29)_ = 2.50, disgusted *t*_(29)_ = 3.46, or sad faces *t*_(29)_ = 4.30, all *p* < 0.02. There was no difference in priming scores for angry faces compared to disgusted *t*_(29)_ = 0.99, or sad faces *t*_(29)_ = 1.71, both *p* > 0.05. Nor was there a difference between priming scores for disgusted and sad faces, *t*_(29)_ = 0.51, *p* > 0.05. In contrast, follow-up analyses revealed no significant main effect of emotion for the MDD group, *F*_(3, 48)_ = 2.35, *p* > 0.05, η^2^ = 0.13, suggesting that MDD participants did not experience differential priming for happy faces compared to negative faces.

## Discussion

The current study examined the effect of emotion on cognitive processing in MDD using an affective priming task with prolonged presentation of facial expressions of emotion. Participants displayed *reverse* priming for angry, disgusted, and happy expressions: participants took longer to identify emotional faces when they were preceded by an emotional face of the same valence vs. preceded by a neutral face. In addition, our analysis yielded a significant valence by group interaction. CTLs showed greater reverse priming from happy compared to angry, disgusted, and sad facial expressions; however, no such valence effect was obtained in the MDD group. Results suggest that the emotionality of the prime affected cognition in the control group. More specifically, when CTL participants viewed a happy face first, it took them longer to process the subsequent happy face. Longer reaction times could be viewed as evidence of “impaired” cognition; however, when longer reaction times indicate more time is spent processing positive expressions, it might actually be functional. In fact, dwelling on positive facial expressions may enhance mood (e.g., Vrugt and Vet, 2009). With this in mind, the fact that positive expressions are not associated with prolonged cognition in the MDD group might impair their ability to use positive expressions to recover from negative mood states.

Although findings of reverse priming are not consistent with our hypotheses, reverse priming effects have been reported in several other studies (e.g., Dannlowski et al., 2006b; Klauer et al., 2009). When interpreting priming results, Bargh and Chartrand (2000) emphasize the importance of considering the delay between prime and target presentation. With brief delays between prime and target (i.e., less than 250 ms), Bargh and Chartrand suggest that only automatic effects should influence people's response to the target. The prime should therefore facilitate faster responses to congruent targets, as is traditionally observed in priming studies. As the delay between prime and target increases, however, strategic and elaborative processing of the prime is expected to override traditional priming effects. Thus, the more difficulty people have disengaging from the prime, the slower they will be to respond to the target. Supporting this, Klauer and colleagues documented reverse priming effects using a prime-target delay of 420 ms (Klauer et al., 2009), and they suggest similar reverse priming effects would also be observed for prime-target delays between 300 and 600 ms. In the current study, 500 ms separated the presentation of the prime and target, which could have allowed attentional capture and elaborative processing of the prime. Moreover, other studies note the contribution of affectively extreme primes to reverse priming effects (e.g., Glaser and Banaji, 1999; Dannlowski et al., 2006b). Our use of facial expressions displaying 100% emotional intensity may have further encouraged attentional capture. Reverse priming effects, therefore, likely reflect people's difficulty disengaging from angry, happy, and disgusted facial expressions captured by attention.

In addition, our findings demonstrate greater reverse priming for happy vs. negative facial expressions in the CTL group, suggesting that CTLs had particular difficulty disengaging from positive compared to negative facial expressions. In contrast, the MDD group showed no effect of prime valence. Our findings are in line with a growing literature showing that non-depressed participants show preferential processing of positive material that is not present in participants with depression (e.g., Deveney and Deldin, 2004; Gotlib et al., 2011). Attention capture by positive information and difficulties disengaging from positive material in the CTL group may contribute to resilience and to the ability to repair negative affect (Joormann et al., 2010) and may therefore represent an important “cognitive vaccine” against depressed mood (Holmes et al., 2009). In contrast, the lack of this “vaccine” in the MDD group might place individuals at increased vulnerability to experience prolonged negative mood states. Finding from the current study also dovetail with results from recent neuroimaging research. For example, Surguladze et al. (2005) found that CTL—but not participants with depression—demonstrated linear increases in limbic-subcortical and extrastriate visual object processing regions when processing happy facial expressions. The authors make important links between neural processing differences and attentional biases typical of depression (see Mathews and MacLeod, 2005, for a review). Similarly, whereas participants with depression showed greater amygdala response to implicitly presented sad faces, CTL showed greater amygdala response to implicitly presented happy faces (Victor et al., 2010), further highlighting that healthy but not depressed individuals show a processing bias toward positive stimuli. Research also has demonstrated associations between anhedonia and neural response to happy expressions (e.g., Keedwell et al., 2005). More specifically, anhedonia severity was associated with greater activity in the ventromedial prefrontal cortex and less activity in the amygdala/ventral striatal regions when processing happy expressions. Future research might therefore examine whether there is a similar correlation between anhedonia severity and degree of priming effects for happy faces.

Several limitations in the current study should be noted. Given the small sample size, it is possible that the follow-up tests were underpowered to detect differences between the CTL and MDD groups. Null findings should therefore be interpreted with caution and replication of the current findings within a larger clinical sample is warranted. That the group by emotion interaction was significant despite a potential lack of power, however, speaks to the strength of this finding. Second, some participants in the MDD group met criteria for a comorbid diagnosis. Because the sample size in the current study prevented us from examining the effects of comorbidity, future research might consider an a priori examination of the role of comorbid anxiety disorders.

Despite these limitations, the current study provides an important look at participants' disengagement from emotional facial expressions captured by attention. CTLs had difficulty disengaging from happy compared to negative facial expressions. Prolonged processing of positive affect might help people regulate negative emotions or “vaccinate” them against future negative mood (e.g., Holmes et al., 2009; Joormann et al., 2010). Moreover, the bias toward positive facial expressions could facilitate prolonged engagement in positive social interactions, which has also been shown to protect individuals against negative mood (e.g., Paykel, 2007). The fact that individuals with MDD failed to show this protective positive bias might therefore contribute to the onset and maintenance of depressive episodes.

### Conflict of interest statement

The authors declare that the research was conducted in the absence of any commercial or financial relationships that could be construed as a potential conflict of interest.
